# The COCCOS study: A protocol for a multicentric quasi-experimental study of a healthcare transition program for young people with chronic conditions

**DOI:** 10.1371/journal.pone.0353932

**Published:** 2026-08-17

**Authors:** Lisa Van Wilder, Natwarin Janssens, Kim Van Hoorenbeeck, Karsten Vanden Wyngaert, Eva Goossens, Delphine De Smedt

**Affiliations:** 1 Department of Public Health and Primary Care, Faculty of Medicine and Health Sciences, Ghent University, Ghent, Belgium; 2 Department of Nursing and Midwifery, Faculty of Medicine and Health Sciences, University of Antwerp, Antwerp, Belgium; 3 Department of Pediatrics, Antwerp University Hospital, Antwerp, Belgium; 4 Laboratory of Experimental Medicine and Pediatrics (LEMP), University of Antwerp, Antwerp, Belgium; 5 Ghent University Hospital, Ghent, Belgium; 6 Department of Patient Care, Antwerp University Hospital, Antwerp, Belgium; PLOS: Public Library of Science, UNITED KINGDOM OF GREAT BRITAIN AND NORTHERN IRELAND

## Abstract

**Introduction:**

The growing number of adolescents and young adults (AYAs) living with chronic conditions highlights the need for effective healthcare transition programmes (TPs) to support the transfer from paediatric to adult care. Despite existing recommendations, many AYAs experience inadequate preparation, resulting in disrupted care and adverse health outcomes. The COCCOS project developed a co-designed TP to address these challenges. This study presents the protocol for an evaluation of this programme.

**Methods and analysis:**

A multicentric quasi-experimental pretest–posttest study will be conducted in two Belgian university hospitals. AYAs aged 17–18 years with type 1 diabetes, asthma, or obesity will be recruited into an intervention group receiving the COCCOS TP or a control group receiving usual care. The intervention consists of four structured consultations over 13–16 months, focusing on transition preparation, autonomy, and continuity of care. Primary outcome is transition readiness, measured using the Transition Readiness Assessment Questionnaire. Secondary outcomes include patient empowerment, medication adherence, health-related quality of life, and mental health. Data will be collected at baseline, transfer, and four months post-transfer. Cost-effectiveness will be assessed from a healthcare payer perspective using quality-adjusted life years. A mixed-methods process evaluation will examine implementation fidelity, acceptability, and contextual factors through observations, surveys, and interviews. The study is registered on ClinicalTrials.gov under identifier NCT07145671.

**Discussion and conclusion:**

This study will provide comprehensive evidence on the feasibility, preliminary effectiveness, cost-effectiveness, and implementation of a co-designed TP in real-world clinical practice. By integrating clinical, economic, and process outcomes, it addresses key gaps in transitional care research. Findings will inform refinement of the programme and the design of a future large-scale trial, contributing to the development of sustainable, patient-centred transition care for AYAs with chronic conditions.

## 1. Introduction

The rising prevalence of chronic conditions poses a growing challenge to health systems worldwide. Over the past decade, the number of adolescent and young adults (AYAs) living with a chronic condition has grown, with an estimated 10–25% affected, of whom over 85% have the prospect of surviving into adulthood [1–3]. This shift is driven by both an increasing prevalence of childhood-onset conditions, such as asthma, obesity, and type 1 diabetes, associated with environmental, behavioural, and diagnostic factors, as well as improved survival rates among children with congenital conditions, including cystic fibrosis and congenital heart disease [3].

Despite improved survival rates, these individuals often continue to carry a lifelong disease burden and an increased risk of complications, requiring continuous and specialized care across the life course [4,5]. Given that healthcare needs change considerably across the lifespan, AYAs are required to transition from paediatric to adult-oriented care upon reaching adulthood [4,5]. This process requires timely developmental and practical preparation, yet many patients transfer before being adequately prepared [4,5]. Indeed, approximately half of AYAs and their families do not receive sufficient support for this transfer [6]. Adolescence is a particularly vulnerable period, characterized by psychosocial and physical changes that may reduce treatment adherence and increase risk-taking behaviours [7,8]. As a result, up to 40% of AYAs experience disrupted care after transfer to adult services [9,10], often due to differences in care organization, including reduced interdisciplinary support, greater emphasis on patient autonomy, and limited expertise in childhood-onset conditions and the specific needs of young adults [11]. Such disruptions may lead to adverse outcomes such as worsening health status, treatment non-adherence, discontinuation of care, increased hospitalizations and emergency department visits, and higher healthcare costs [12–15].

Structured healthcare transition programmes (TPs) are, therefore, recommended to support a gradual and well-prepared transition towards adulthood and adult care. By fostering self-management skills, improving treatment adherence, and empowering patients, TPs aim to prevent health deterioration and may reduce healthcare costs by 3–14% [16]. Although several guidelines highlight the importance of transitional care, important knowledge gaps in real-world data and effectiveness remain [5,14], particularly regarding which programme elements are most beneficial in specific contexts and populations, process-level factors such as uptake and implementation, and experiences of patients and caregivers [15,17]. Recent consensus statements from the American and European paediatric societies call for more research, especially on common conditions like asthma and childhood obesity [14,18]. While empirical data support the clinical effectiveness of TPs, their long-term sustainability within everyday routine hospital care pathways remains limited [19].

Building on the first work package of the COCCOS project, which developed a TP using an experience-based co-design (EBCD) methodology [20,21], this study presents the protocol for the second phase: a multicentric quasi-experimental study. The study aims to provide insights into the program’s potential impact on clinical and patient-reported outcomes, its cost-effectiveness, and the experiences of patients and healthcare providers with its implementation.

## 2. Methods

### 2.1. Study design

A multicentric quasi-experimental pretest-posttest study was designed to evaluate a healthcare transition programme in AYAs with chronic conditions. This design enables the assessment of outcomes over time within an intervention cohort, while also allowing comparison with a cohort receiving standard care. The quasi-experimental approach was selected to account for the practical constraints of real-world clinical settings, where randomization is often neither feasible nor appropriate, including limitations related to time investment for patients and healthcare providers, potential burden of repeated measurements, reduced feasibility of frequent follow-up in the control group, and an increased risk of attrition in a fully randomized design. In addition to evaluating effectiveness, this design supports the assessment of implementation processes and the experiences of both patients and healthcare providers, thereby providing a comprehensive evaluation of the intervention in routine care practice in Flanders (Belgium). At the time of manuscript submission, the study is ongoing. Participant recruitment took place between March 2025 and March 2026. Data collection will continue until December 2026, allowing for follow-up of enrolled participants according to the study protocol. Data analysis is anticipated to be completed by March 2027, with study results expected to be reported in 2027. The study is registered on ClinicalTrials.gov under identifier NCT07145671. More details can be found at: https://clinicaltrials.gov/study/NCT07145671?cond=Coccos&viewType=Card&rank=1.

### 2.2. COCCOS transition programme

**Fig 1:**
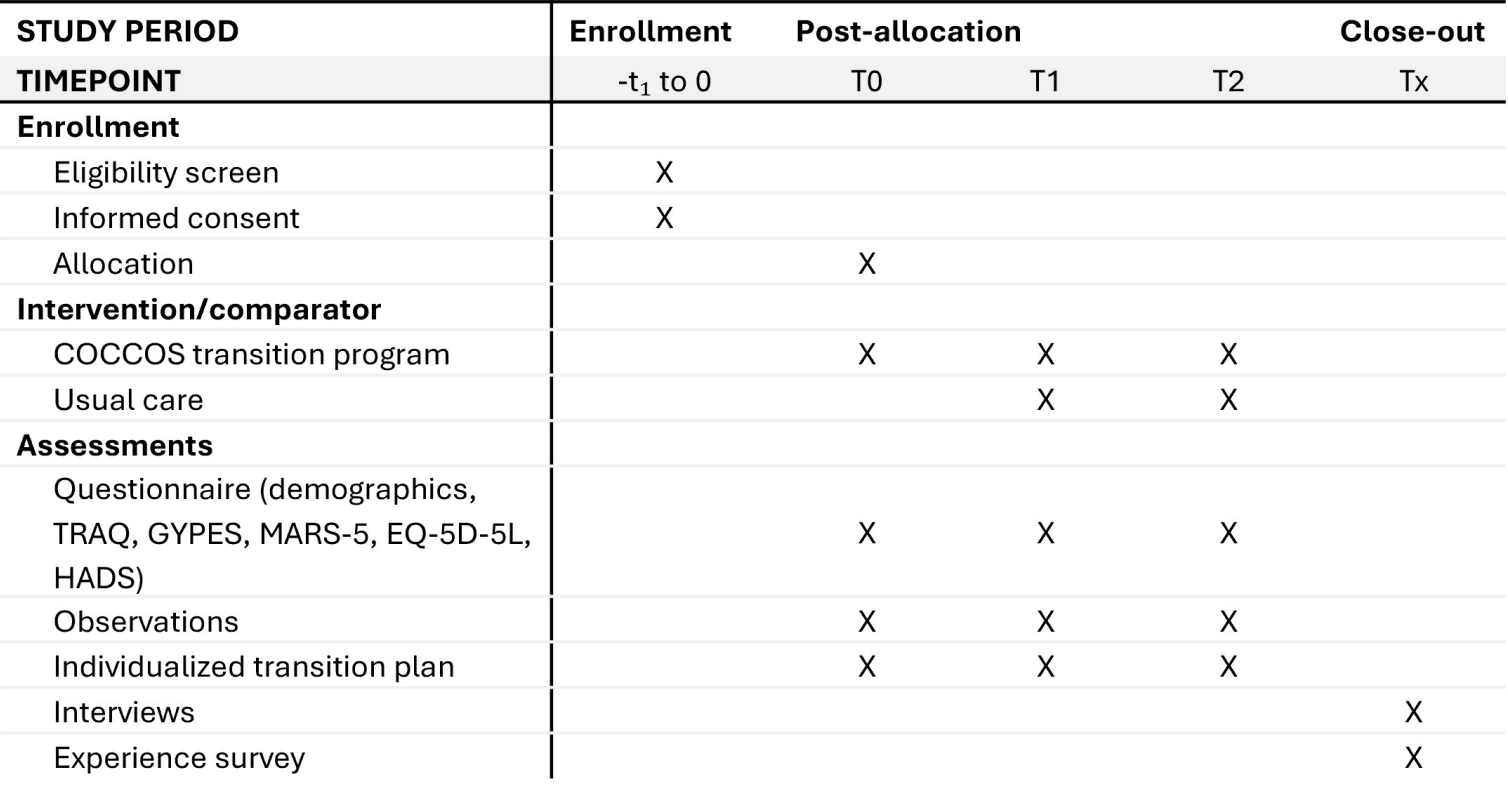
SPIRIT checklist.

The TP was developed within the first work package of the COCCOS project, in close collaboration with key stakeholders, including AYAs, parents, and healthcare providers. A participatory action research approach was adopted, incorporating methods such as EBCD and photovoice [20,22]. Detailed information on the development and content of the TP has been described previously [21]. Fig 1 presents the SPIRIT checklist for this research.

**Fig 2:**
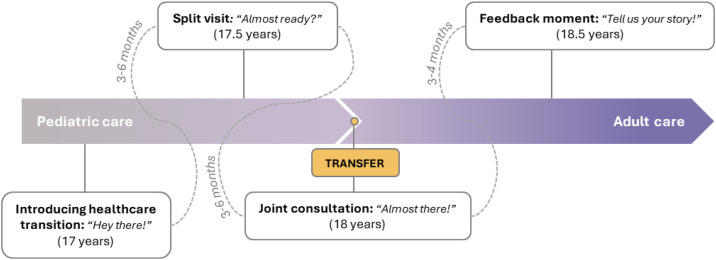
Overview of the four key moments in the COCCOS transition programme.

In brief, the TP consists of four key components delivered over a period of 12–16 months, depending on pathology-specific care trajectories (see Fig 2):

(1) **Key moment 1: Introducing healthcare transition *“Hey there!”* (17 years):** This initial, paediatric care-led consultation introduces the concept and trajectory of healthcare transition to the AYA and their family. The timing of transfer to adult care is determined through shared decision-making. A paediatric healthcare provider is appointed as the primary contact person – preferably someone already trusted by the AYA – and can be contacted for any transition-related concerns. In addition, AYAs are encouraged to identify a contact person within their personal support network. All agreements and relevant information are documented in an individualized transition plan. AYAs receive brochures and flyers containing information on healthcare transition, including what the transition entails, the key differences between paediatric and adult care services, and details about their future adult-care team.
(2) **Key moment 2: Split visit *“Almost ready?”* (17.5 years):** At least one independent consultation takes place between the introductory and joint consultation. During this visit, parents or family members may be asked to leave part of the consultation to encourage autonomy and direct communication between the AYA and the healthcare provider. Prior to the consultation, AYAs are asked to complete the Ready Steady Go checklist [23], which serves as a structured tool to assess transition readiness. The individualized transition plan is reviewed and updated accordingly, and any concerns raised by the AYA are addressed by the paediatric team.
(3) **Key moment 3: Joint consultation *“Almost there!”* (18 years):** The final consultation before transfer is conducted jointly by paediatric and adult healthcare providers. This consultation facilitates continuity of care and allows the AYA to become acquainted with the adult care team. A contact person within the adult care setting is appointed. The Ready Steady Go-checklist is completed beforehand to guide the consultation and ensure that key transition domains are addressed.
(4) **Key moment 4: Feedback moment *“Tell us your story!*” (18.5 years):** Approximately three to four months after transfer, preferably after the first adult care consultation, the COCCOS researcher contacts the AYA to evaluate the transition process. The Ready Steady Go-checklist is completed beforehand to support reflection. Feedback is communicated to both paediatric and adult care teams, and any arising issues are addressed through additional support, referrals, or extra consultations if needed.

The intervention will be delivered during routine outpatient consultations with the paediatrician, supported by structured guidance from the research team, including preparatory contact before each consultation. Its design and delivery are based on insights from the EBCD process, which highlighted the need for clear structure, consistency across providers, and practical tools to support transition discussions. Prior to each consultation, the paediatrician will be contacted by the researcher and informed about the specific component of the intervention to be delivered at that visit, ensuring timely preparation and alignment with the study protocol.

To facilitate implementation, the research team will provide a paper folder containing all intervention materials, including the Ready Steady Go-checklists, the individualized transition plan, and informative brochures and flyers. The folder is organized in a stepwise manner, corresponding to the sequence of consultations, and includes a checklist with concrete action points for each visit. These checklists specify the key elements to be addressed, such as topics to discuss, documents to complete, and actions to be taken with the adolescent and their family. These standardised implementation tools support the paediatrician in delivering the intervention as intended, while reducing variability between providers and centres. In addition, the use of standardized materials and checklists promotes intervention fidelity and completeness, while still allowing flexibility to tailor discussions to the individual needs of the adolescent. The integration of the intervention into routine consultations minimizes additional burden on both patients and healthcare providers, thereby enhancing feasibility and facilitating potential future implementation in standard care.

### 2.3. Setting, participants, and recruitment

#### 2.3.1. Setting.

**Fig 3:**
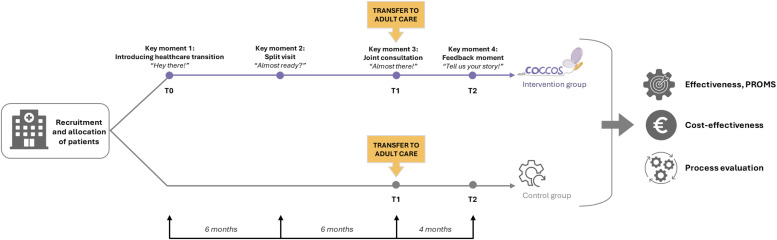
Overview of the quasi-experimental pre-test post-test study.

The study will be conducted at the Queen Mathilde Mother and Child Center, Antwerp University Hospital (UZA), and the Princess Elisabeth Pediatric Hospital, University Hospital Ghent (UZ Ghent). The TP will be implemented over a total period of 16 months, including a post-transfer assessment (see Fig 3).

Participants in the intervention group will receive the TP over 13–16 months, depending on the trajectory specific to their condition. Questionnaire assessments will be administered at three time points: at baseline, prior to the start of the intervention (T0); at the time of transfer to adult care services (T1), with the questionnaire completed immediately after the last consultation on paediatric care services; and four months post-transfer (T2). For the control group, whom receive usual care, assessments will be conducted at the time of transfer (T1), with the questionnaire completed immediately after the last consultation on paediatric care services, and four months post-transfer (T2).

#### 2.3.2. Participants.

The study will focus on three common chronic childhood-onset conditions: type 1 diabetes, asthma, and obesity. The three diagnostic groups will be included to ensure variability and representativeness. Eligibility criteria for the intervention group include: being 17 years old, having a diagnosis of one of the target conditions, at least one paediatric outpatient visit in the past 12 months, Dutch-speaking, and a planned transfer to adult care within 12 months. Participants in the control group are aged 17,5–18 years, Dutch-speaking, with a planned transfer to adult care in the near term. Patients with severe mental, cognitive, and/or neurological impairments are excluded. At UZ Ghent, only type 1 diabetes patients will be included, while at UZA, patient with asthma and obesity patients will be included.

Based on reference data from previous studies, the intervention target was defined as an improvement in the primary outcome corresponding to a one standard deviation (SD) increase in the TRAQ score [24–26]. This target was set assuming 90% power, an effect size of d < 0.6, and a significance level of α = 0.05. A power analysis conducted using G*Power 3.0 indicated that 22 participants per group (intervention and control) would be required. Considering the heterogeneity of the AYA population and comparable disease complexity across subgroups, this sample size was applied at the population level, resulting in a total required sample of 44 participants. To account for an anticipated attrition rate of 25% over a 24-month follow-up period [27], as well as potential recruitment challenges, the total target sample size was increased to 55 participants. Accordingly, a minimum of 28 participants per group (intervention and control) should be recruited.

For the process evaluation, all participating AYAs, their families, and involved healthcare providers will be invited to complete quantitative assessments. A purposive subsample will be selected for qualitative evaluation to capture variability in experiences and perspectives, ensuring representation across conditions, sites, and roles in the care process.

#### 2.3.3. Recruitment.

Recruitment of AYAs will be facilitated through paediatricians at the participating hospitals. Potentially interested paediatric care teams will first be identified using convenience sampling, based on their prior involvement in the initial COCCOS work package. These teams will be invited to attend an informational session during their regular team meetings, where researchers will present the study objectives and procedures, address questions, and invite teams to participate. Participating paediatricians, or their hospital/department administrative staff, will subsequently identify eligible patients based on predefined inclusion criteria. Eligibility will primarily be determined by the age of the AYA, which will also define allocation to either the intervention or control group based on predetermined age criteria. This pragmatic approach reflects real-world transition practices, where transfer to adult care is primarily age-driven. Although this may introduce potential selection bias due to age-related differences between groups, it enhances the ecological validity of the study by mirroring routine care pathways. During routine consultations, paediatricians will briefly introduce the study to eligible AYAs and their parents and invite them to participate. AYAs who express interest will be referred to a member of the research team present at the hospital department. The researcher will provide detailed written and verbal information about the study, answer any remaining questions, and guide both the AYA and their parents through the informed consent process.

### 2.4. Data collection

#### 2.4.1. Effectiveness.

A questionnaire is developed based on the existing literature and includes several validated instruments:

**Transition Readiness Assessment Questionnaire (TRAQ), version 6.0.** As the primary aim of the TP is to prepare AYAs for transition to adult care, the primary outcome is transition readiness. This is assessed using the TRAQ (version 6.0) [25,26,28]. The TRAQ is a 20-item self-report instrument designed to evaluate the skills necessary for a successful transition. It covers four domains: (1) medication management, (2) appointment keeping, (3) tracking health issues, and (4) talking with providers. In addition, it includes two global transition readiness items. Items are scored on a 5-point Likert scale, with total scores ranging from 0 to 53, where higher scores indicate greater transition readiness [28]. Prior to use in this study, the TRAQ will be forward-backward translated into Dutch (Flemish) to ensure linguistic and cultural validity (REF TRAQ).

**Gothenburg Young Persons Empowerment Scale (GYPES).** Patient empowerment is assessed using the GYPES [29], a validated 15-item instrument specifically developed for young people with chronic conditions. The instrument captures multiple domains of empowerment, including knowledge and understanding of one’s condition, personal control, identity, shared decision-making, and the ability to enable others. Responses are scored on a 5-point Likert scale, with higher scores reflecting greater levels of empowerment. The GYPES provides insight into how confident AYAs feel in managing their health and engaging in care.

**Medication Adherence Report Scale (MARS-5).** Medication adherence is evaluated using the MARS-5 [30,31], a brief 5-item measure assessing both intentional and unintentional non-adherent behaviours. Items address behaviours such as forgetting to take medication or deliberately altering doses. Each item is rated on a 5-point Likert scale, with higher scores indicating better adherence. The MARS-5 is widely used due to its simplicity and sensitivity to different types of non-adherence.

**EQ-5D-5L.** Self-perceived health status is measured using the EQ-5D-5L [32], a standardised instrument covering five dimensions (mobility, self-care, usual activities, pain/discomfort, and anxiety/depression). Each dimension is rated across five levels of severity, ranging from no problems to extreme problems. Responses define a specific health state that can be converted into a utility score using country-specific value sets. Additionally, it includes a visual analogue scale (VAS), where AYAs to rate their overall health on a scale from 0 (worst imaginable health) to 100 (best imaginable health).

**Hospital Anxiety and Depression Scale (HADS).** Symptoms of anxiety and depression are assessed using the HADS [33], a 14-item instrument designed for use in clinical populations. It consists of two subscales: anxiety (seven items) and depression (seven items). Each item is scored on a 4-point scale, resulting in subscale scores ranging from 0 to 21. Higher scores indicate higher levels of anxiety or depressive symptoms. The HADS is commonly used because it focuses on psychological symptoms while minimizing the influence of physical illness.

At baseline, data will be collected using a paper-based questionnaire. This approach allows the researcher to be present during completion, providing clarification and support in case participants experience difficulties or have questions. At subsequent time points, questionnaires will be administered electronically via a secure online survey platform (i.e., REDCap) and distributed through an automated email system. To maximize response rates, reminder emails will be sent to participants who have not completed the questionnaire within a predefined timeframe. The questionnaire was pilot tested internally by the research team to evaluate its feasibility and acceptability. Specifically, the pilot evaluation focused on readability, comprehensibility of the items, and overall completion time. On average, completion of the questionnaire takes approximately 10–15 minutes, indicating an acceptable respondent burden.

#### 2.4.2. Cost-effectiveness.

Costs related to the implementation of the TP will be systematically collected. These will include personnel costs associated with the time investment of healthcare professionals involved in the program (e.g., physician, nurses, psychologists, dieticians, and diabetes educators), as well as costs related to multidisciplinary team meetings. In addition, material costs will be considered, including printed educational materials and other resources used within the intervention. Healthcare resource use will be estimated using published data on healthcare utilization patterns, combined with unit costs derived from the National Institute for Health and Disability Insurance (NIHDI) nomenclature of healthcare services. This approach will allow estimation of healthcare costs associated with outpatient visits, hospitalizations, and other reimbursed services. Health outcomes will be derived from the EQ-5D-5L utility values. Together with cost data and transition probabilities, these will serve as input to the model. Quality-adjusted life years (QALYs) will be calculated as an outcome of the analysis and, together with costs, will form the basis for the TP’s cost-effectiveness evaluation.

#### 2.4.3. Process evaluation.

AYAs, families, and healthcare professionals who participated in the TP will be invited to a process evaluation, aimed at assessing both the implementation of the TP and how it was received, in accordance with the Medical Research Council guidance [34]. This evaluation is essential to contextualize findings from the effectiveness study, helping to explain why and when the TP was or was not successful. Several programme dimensions will be examined, including: intervention fidelity (the extent to which the TP was delivered as intended with consistent quality), dose (the extent to which the TP was delivered by healthcare providers and received by patients and families), reach (the proportion and characteristics of patients who participated), context (factors that facilitated or hindered TP uptake), and change (changes made by care team during implementation) [35]. Data will be collected both during and at the end of the programme using a mixed-method approach, combining quantitative and qualitative data to enable triangulation and provide comprehensive coverage of all program dimensions:

**Observations.** Longitudinal non-participatory observations of outpatient visits will be conducted using a structured checklist to assess programme fidelity across three intervention components (key moments 1–3). For each chronic condition, two observations per measurement moment will be conducted in the intervention group (total n = 18), ensuring variation across hospital settings, consultation type, and participant characteristics.

**Individual transition plans.** Data from the individualized transition plans will be extracted to evaluate fidelity, participant engagement, adherence, and overall quality of programme delivery, including dose delivered and dose received.

**Interviews.** Semi-structured interviews (15–30 minutes) will be conducted at the end of the intervention to explore experiences, perceived facilitators and barriers, and contextual factors. A total of five AYAs will be interviewed, including four from the intervention group (representing the three diagnostic groups) and one from the control group. In addition, five healthcare professionals and three parents will be interviewed. Interview data will be used to further explore programme delivery and uptake, as well as contextual influences affecting implementation. An overview of the interview topic guide can be found in Table 1.

**Table 1:** Topic guide that will be used for the process evaluation of the COCCOS intervention.

Topics	Main questions
**Adolescents and young adults (AYAs)**	
*Program experience*	- Can you describe your overall experience with the transition program, including the contact moments (content and frequency)?
*Use of tools and information*	- Did you use your individualized transition plan outside of consultations? What was your experience with it? - What do you remember from the information brochures, and was any important information missing?
*Preparedness for transition*	- To what extent did you feel prepared for the transition to adult care? Why/why not?
*Evaluation and improvement*	- How would you rate the program (0–10) and why? What improvements would you suggest?
**Parents**	
*Program experience*	- Can you describe your overall experience with the transition program, including the contact moments?
*Communication and support*	- Did you feel there was sufficient opportunity to address your and your child’s concerns and questions?
*Use of tools and information*	- Did you or your child use the individualized transition plan outside consultations? What was your experience? What do you remember from the information materials, and was anything missing?
*Impact and preparedness*	- Did you notice any changes in your child during the transition? Did you feel prepared for the transition to adult care? Why/why not?
*Evaluation and improvement*	- How would you rate the program (0–10) and why? What improvements would you suggest?
**Healthcare professionals**	
*Experience with the program and protocol*	- Can you describe your experience with implementing the transition program and its protocol? Which components were most useful or less feasible?
*Organization and collaboration*	- How would you describe coordination within the team and collaboration between pediatric and adult care?
*Use of tools in practice*	- What is your experience with the use of the individualized transition plan?
*Barriers, facilitators, and feedback*	- What factors facilitated or hindered implementation? What feedback did you receive from patients/families, and how was it used?
*Support, evaluation, and sustainability*	- Did you feel sufficiently supported? How would you rate the program (0–10) and why? Is it feasible to implement sustainably in your service?

**Experience survey.** An experience questionnaire, including a brief set of sociodemographic items (e.g., age, sex, socio-economic status), will be administered electronically to AYAs (all participants), family members (where contact details are available), and healthcare professionals (all) at the end of the intervention to assess perceptions of the TP and completion of intervention components. Survey data will be used to assess dose received, operationalized as satisfaction with the intervention. In addition, the results will inform and guide the interviews, with certain topics explored in greater depth during the interviews. Table 2 provides an overview of the questions included in the experience survey.

**Table 2:** Overview of the questions included in the experience survey for the process evaluation of the COCCOS intervention.

Section	Content questions	Response format
**Adolescents and young adults (AYAs)**		
*Socio-demographic information*	- Age - Gender - Nationality - Parental nationality - Chronic condition	- Years - Male/female/other - Belgian/other - Belgian/other - Obesity/asthma/type 1 diabetes
*Contact moments*	- Which contact moments did you attend? - Why did you not attend certain contact moments? - Did you complete the Ready Steady Go-checklist? - Did the contact moments teach you something? - What do you remember most? - Did you feel prepared for transition after the joint consultation? - What could be improved?	- Categorical - Open-ended - Categorical + open-ended - Categorical + open-ended - Open-ended - Open-ended - Open-ended
*Individualized transition plan*	- Did you consult the individualized transition plan outside consultations? - How easy/difficult did you found the individualized transition plan?	- Categorical + open-ended - Likert scale
*Information brochures*	- Which brochures did you receive? - Did you read the brochures?	- Categorical - Categorical + open-ended
*General evaluation*	- How satisfied were you with the COCCOS intervention? - Would you recommend the programme?	- Likert scale - Categorical + open-ended
**Parents**		
*Socio-demographic information*	- Age - Gender - Nationality - Child’s chronic condition	- Years - Male/female/other - Belgian/other - Obesity/asthma/type 1 diabetes
*Contact moments*	- Which contact moments did your child attend? - Why were certain contact moments not attended? - Did your child complete the Ready Steady Go-checklist? - Did the contact moments teach your child something? - Did the contact moments inform you as a parent? - Did you feel prepared for transition? - What could be improved?	- Categorical - Open-ended - Categorical + open-ended - Categorical + open-ended - Categorical + open-ended - Categorical + open-ended - Open-ended
**Healthcare professionals**		
*Socio-demographic information*	- Gender - Profession - Years of experience	- Male/female/other - Open-ended - Years
*General evaluation*	- Was the programme manual sufficient? - Was the individualized transition plan discussed during consultations? - Did contact moments add value for AYAs and parents? - Were AYAs and parents prepared for transition? - How easy/difficult did you found the individualized transition plan? - How easy/difficult did you found the brochures? - How satisfied were you with the COCCOS intervention? - Would you continue implementing the programme? - Do you have additional suggestions?	- Categorical + open-ended - Categorical + open-ended - Categorical + open-ended - Categorical + open-ended - Likert scale - Likert scale - Likert scale - Categorical + open-ended - Open-ended

**Registration form.** Recruitment of hospital departments and eligible patients will be monitored using a registration form, with reasons for non-participation documented where possible to inform assessment of recruitment processes and programme reach.

### 2.5. Data analysis

#### 2.5.1. Effectiveness.

Baseline characteristics will be summarized using appropriate descriptive statistics (e.g., means and standard deviations or medians and interquartile ranges for continuous variables, and frequencies and percentages for categorical variables). Within the intervention group, pre-post changes over time will be evaluated using paired t-tests or non-parametric equivalents when assumptions are not met. Given the non-randomized and exploratory nature of the study, analyses comparing the intervention and control group will primarily be descriptive and exploratory. Potential baseline differences between groups will be taken into account by exploring propensity score-based approaches, such as inverse probability of treatment weighting (IPTW), if deemed feasible given the sample size and data distribution. Outcome differences between groups will be interpreted cautiously, with emphasis on effect sizes and confidence intervals rather than formal hypothesis testing alone. All statistical tests will be two-sided, and statistical significance will be set at p < 0.05. Missing data will be reported and handled transparently; given the expected small sample size, no multiple imputation procedures are planned.

#### 2.5.2. Cost-effectiveness.

A cost-effectiveness model will be developed following ISPOR guidelines for trial-based economic evaluations [36]. The model will evaluate the potential economic value of the TP compared to standard care from a healthcare payer perspective. While a societal perspective may capture broader costs and consequences, such as productivity losses and informal care, it is not the primary perspective for decision-making in this context. The analysis will adopt a time horizon sufficient to capture all relevant costs and health outcomes associated with the intervention, extending beyond the trial follow-up period where necessary. Health outcomes will be expressed in terms of QALYs, integrating both quality and quantity of life, based on directly measured health state utilities obtained from the EQ-5D-5L utility scores. These intermediate outcomes will help capture potential longer-term benefits of the intervention beyond trial follow-up period. Costs will include healthcare resource use as well as the operational costs of implementing the TP. The primary outcome of the model will be the incremental cost-effectiveness ratio (ICER), expressed as the additional cost per QALY gained.

Future costs and health outcomes will be discounted in accordance with Belgian guidelines, applying a discount rate of 3% for costs and 1.5% for health effects [37]). Uncertainty in the ICER will be explored through both deterministic (one-way) sensitivity analyses and probabilistic sensitivity analysis. A cost-effectiveness acceptability curve will be generated to assess the probability of cost-effectiveness across a range of willingness-to-pay thresholds. In addition, value of information analyses, including the expected value of perfect information and expected value of partial perfect information, will be conducted to quantify decision uncertainty and inform the potential value of further research [38].

#### 2.5.3. Process evaluation.

Quantitative data (including enrolment registration forms, sociodemographic variables, and experience surveys) will be analysed using descriptive statistics. Information extracted from the individualized transition plans will be further examined through quantitative content analysis [39]. Interviews will be audiotaped and transcribed verbatim, and qualitative data will be analysed thematically using NVivo software [40]. All data sources will be systematically organized according to the stages of the transition process (i.e., pre, peri, and post) and key domains of interest (e.g., readiness, support, engagement). This structured framework allows quantitative and qualitative data to be directly linked, enabling triangulation and comparison across sources. Integrating data in this way ensures that findings from one method can contextualize and enrich those from another, providing a full and nuanced understanding of the programme’s impact.

### 2.6. Ethics approval

The study follows a multicentric design and has received ethical approval from the Ethical Committee of Antwerp University Hospital and Ghent University Hospital (reference number: B3002024000166), Belgium.

Written informed consent will be obtained from all participants, and assent will be sought from minors where applicable. Participants will be informed that refusal to participate, or withdrawal from the study at any time, will have no impact on their current or future care.

All data will be securely stored in de-identified form and will only be accessible to authorized project researchers via encrypted platforms with two-factor authentication. Data will be retained for a period of 20 years.

Study findings will be disseminated through peer-reviewed publications and presented at national and international conferences. In addition, a summary of the results will be shared with clinicians and policy makers through relevant local, national, and international networks and events.

## 3. Discussion

This study presents the protocol of a multicentric quasi-experimental study evaluating the COCCOS TP for AYAs with chronic conditions. By combining assessments of effectiveness, cost-effectiveness, and implementation, the study aims to generate insights on both outcomes and the delivery of a co-designed TP in real-world clinical settings, addressing the need for evidence-based transitional care.

A key strength of this study is its broad evaluation approach, addressing not only clinical but also economic and implementation outcomes [41]. While previous studies have shown potential benefits of structured TPs, such as improved adherence, self-management, and reduced healthcare utilization, evidence is often fragmented and limited to single outcomes or short-term effects [42–44]. By integrating measures of transition readiness, empowerment, treatment adherence, mental health, and health-related quality of life, this study calls for more comprehensive evaluations. The inclusion of a cost-effectiveness analysis is particularly relevant given increasing pressure on healthcare systems to allocate resources efficiently. Demonstrating the economic value of TPs is essential to support their long-term integration into routine care pathways. Another important strength is the participatory development of the intervention. The TP was co-designed with AYAs, families, and healthcare providers using EBCD methods, enhancing its relevance, acceptability, and feasibility [45]. Compared with top-down designed interventions, co-created programmes better align with patient needs and clinical practice realities, increasing uptake and facilitate implementation in practice [46,47]. However, long-term sustainability is likely to depend not only on co-creation but also on organizational and system-level support. The TP’s structured yet flexible design also allows adaptation to different disease trajectories and care contexts. This study further contributes to the literature by focusing on prevalent chronic conditions, addressing a gap in the literature that, although well-studied in type 1 diabetes, has been less explored for other common conditions and has often focussed on rare or highly specialized conditions [14]. Including multiple conditions enables assessment of whether the TP performs consistently across different clinical contexts and enhances the generalizability and scalability of findings. Given the feasibility nature of this study, findings should be interpreted with caution and are intended primarily to inform the design of a future larger-scale trial rather than to draw definitive conclusions on effectiveness [48,49].

Despite these strengths, several limitations should be acknowledged. First, the quasi-experimental design without randomization may introduce selection bias. To address this, IPTW will be used to balance observed covariates between groups, however, this approach does not account for unobserved confounders [50,51]. Second, the study relies on self-reported measures, which may introduce reporting bias and social desirability effects [52,53]. However, the use of validated instruments such as the TRAQ 6.0, GYPES, MARS-5, EQ-5D-5L, and HADS strengthens the reliability and comparability of findings [54]. In addition, the relatively short follow-up period of four months post-transfer may not fully capture long-term outcomes, such as sustained adherence, disease control, or healthcare utilization patterns [55]; nevertheless, it does fully cover transition’s most critical point of healthcare continuity, i.e., the transfer. The embedded process evaluation, however, will help interpret findings and identify barriers and facilitators. As transition is inherently a longitudinal process, future studies should include longer follow-up periods to evaluate the durability of effects. Due to time constrains within the current project, this was not feasible. Implementation across multiple centres and conditions may also introduce contextual variability, including differences in organizational structures, care practices, and resource availability [56]. While this heterogeneity may complicate interpretation findings, it is also a strength in terms of external validity. The embedded process evaluation will be crucial to understand how contextual factors influence implementation, identify barriers and facilitators, and explain observed outcomes.

Importantly, this study positions TPs within the broader framework of the sextuple aim of healthcare, aiming to improve patient outcomes and experiences, support healthcare providers, optimize costs, and indirectly promote health equity and environmental sustainability [57]. The inclusion of healthcare providers’ perspectives acknowledges that successful implementation depends not only on patient engagement but also on provider workload, interprofessional collaboration, and organizational support.

The findings of this study will have several implications. First, they will inform the refinement and optimization of the COCCOS TP, allowing adaptation of its components based on user feedback and real-world implementation insights. Second, the results will provide key parameters, such as recruitment rates, retention, variability in outcomes, and preliminary effect sizes, needed to design a future larger-scale evaluation study, such as a pragmatic trial or hybrid effectiveness-implementation study. Third, the economic evaluation will offer early evidence to inform policy and decision-making regarding the integration of transition programmes into routine care.

In conclusion, this study addresses critical gaps in the evaluation of transitional care by combining co-designed intervention development with a comprehensive mixed-methods feasibility assessment. By generating evidence on effectiveness, cost-effectiveness, and implementation, it contributes to the development of sustainable, patient-centred transition programmes for AYAs with chronic conditions. Ultimately, improving the healthcare transition from paediatric to adult care has the potential to enhance long-term health outcomes and reduce the burden on healthcare systems.

## Supporting information

S1 File 2. Onderzoeksprotocol COCCOS WP2 3.0_C. (DOCX)

S2 File SPIRIT checklist. (DOCX)

## Abbreviations

AYA: Adolescent and young adult
TP: Transition programme
EBCD: Experience-based co-design
QALY: Quality-adjusted life year
ICER: Incremental cost-effectiveness ratio
