# Supplementary material for: The COCCOS study: A protocol for a multicentric quasi-experimental study of a healthcare transition program for young people with chronic conditions
Source: PLoS One. 2026 Aug 17;21(8):e0353932. doi: 10.1371/journal.pone.0353932 (PMC13480643; doi:10.1371/journal.pone.0353932)
Supplement: Supplementary file 1 [file pone.0353932.s001.docx]

COCCOS study: A study protocol for a feasibility study of a transition program for young persons with chronic conditions

**Central principal investigator**: Prof. dr. Eva Goossens (UAntwerpen/UZA)

**Local principal investigator**: Prof. dr. Ann Van Hecke (UGent/UZ Gent)

**Co-supervisors**: Prof. dr. Delphine De Smedt (UGent), Prof. dr. Kim Van Hoorenbeeck (UAntwerpen/UZA)

**Researchers**: dr. Lisa Van Wilder (UGent), dr. Karsten Vanden Wyngaert (UZ Gent), dra. Natwarin Janssens (UAntwerpen/UGent)

# **LIST OF ABBREVIATIONS**

AYA	Adolescent and young adult

HCP	Healthcare provider

# 1 │BACKGROUND

The rising prevalence of chronic conditions have challenged health systems worldwide. Over the past decade, an increase in prevalence of chronic conditions in youth has been observed ^1^. Approximately 10 to 25% of young patients are affected by chronic conditions, with over 85% of them having the potential to reach adulthood ^2,3^. The origin of this epidemiological shift is two-fold. On the one hand, the incidence of childhood-onset conditions, such as asthma, obesity or type 1 diabetes, increased extensively due to environmental and behavioral changes as well as improved screening and diagnosis. On the other hand, survival rates of children affected by congenital conditions, such as cystic fibrosis or congenital heart disease, improved substantially thanks to medical advancements ^1^. Despite improved survival rates, these young patients are confronted with an amplified disease burden and a life-long risk for complications acquiring specific care needs ^4^. To prevent, treat and manage such complications and comorbidities, continuous specialized care along the life course of patients is recommended ^4,5^. Life-long care should comprehensively address the specific healthcare needs encountered by patients at each respective stage in life (i.e., childhood, adolescence and adulthood). Since the needs faced by adults differ profoundly from those in children, adolescents affected with a chronic condition should be transferred from pediatric care towards a care setting better addressing the medical, psychosocial, and behavioral needs experienced by adults ^4,5^. Furthermore, since new roles, responsibilities and tasks are to be taken up by these patients, as they arrive within adult-focused care, a developmental and practical preparation should be provided. To avoid an unprepared transfer of care, patients should receive guidance and support enabling them to gradually adapt to the set of responsibilities, tasks and expectations encountered in adulthood ^4,5^. It is estimated that about half of chronically ill adolescents and their families do not receive the information needed to achieve a smooth transfer ^6^. Ideally, a gradual preparation of the patient and family, anticipating the eventual transfer to adult care, is provided through the implementation of a structured transition program (TP) (see Figure 1).

**Figure 1. Usual care versus transitional care in young patients with chronic conditions.**

Adolescence is a vulnerable and particularly challenging period, especially in patients suffering from a chronic condition. Because of psychosocial and physical changes, patients are prone to suffer from decreased adherence rates and adverse risk-taking behaviors ^7,8^. Up to 40% of AYAs experience a disruption in access to medical care after their transfer to adult care ^9,10^. An abrupt transfer is found to result in discontinuation of care ^11^, since adult care is characterized by the involvement of multiple care providers, based on a more autonomous patient-physician relationship, but with a less interdisciplinary approach as compared to pediatric care ^12^. As a result, symptom burden and complications related to the disease often develop or accelerate in adolescence and early adulthood, leading to increasing emergency department visits, hospitalizations and healthcare costs ^13^. TPs are essential in the delivery of high-quality healthcare in adolescents with chronic conditions. A TP aims to prevent deterioration of health through the development of self-management skills, increased treatment adherence and patient empowerment, potentially leading to healthcare cost savings between 3 to 14% ^14^. The absence of an adequate TP is found to result in impaired patient health and well-being, medication non-adherence, discontinuation of care, increased emergency department and hospital use, and consequently increased healthcare costs ^15,16^. TPs should ultimately address the quadruple aim of healthcare including enhancing health outcomes, improved patient and healthcare provider experiences, and reduced healthcare costs ^8,17^. Although several recommendations endorsed the need for transitional care in young patients with chronic conditions, several important knowledge gaps remain ^5,15^. To date, the majority of TPs are without clear indications on the uptake, impact or experiences among patients and caregivers ^16,18^. According to recent consensus statements of the American and European Academies of Pediatrics, priority should be given to examining the implementation and effectiveness of TPs in youth with less complex but more prevalent chronic conditions, such as asthma, and childhood obesity ^15,19^. Although empirical data supporting the clinical effectiveness of TPs are present ^20^, in terms of sustainability, TPs do not seem to stay integrated as long-term care pathways in hospitals.

Subsequent to the first work package of the COCCOS project, where a TP has been developed using the Experience-Based Co-Design ^21^, the second study comprises a feasibility study to examine the preliminary impact of the TP in terms of clinical outcomes, cost effectiveness and process indicators.

# 2 │OBJECTIVES

A study is undertaken to examine the feasibility of the newly developed TP in a multicentric setting. Specific objectives for this study are:

1. Examining the impact of the TP in terms of patient-reported health outcomes (PROMs).
2. Examining patients’ and healthcare providers’ experiences related to the TP implementation process.
3. Examining the impact of the TP in terms of cost-effectiveness.

## 3 │METHODS

## 3.1 │Design

A multicentric quasi-experimental, pre-test post-test design will be implemented.

## 3.2 │Intervention

A TP was developed, as part of the first work package of the COCCOS project, in close collaboration with stakeholders (i.e., AYA patients, parents and HCPs) based on participatory action research methods (i.e., Experience-Based Co Design, including photovoice ^22,23^). Based on this study, the developed TP entailed four key moments: (i) a transition consultation, (ii) a consultation without parents, (iii) a joint consultation, and (iv) a feedback moment. Over a course of approximately 9-15 months (this can differ considering different pathologies have different care trajectories (e.g., frequency of consultations)), AYAs will receive an intensive person-centered transition program consisting of the four key moments (see Figure 2). The specific characteristics of each component is defined as followed:

1. **Transition consultation**: The first component of the TP comprises a consultation that is solely focused on the topic of transition, with an introduction of the transition trajectory to the patients and his/her family. This consultation is provided by the pediatric HCP. An introduction to the operational processes performed at the adult care setting will be provided, and the moment of transfer will collectively be decided upon in shared decision-making with the AYA patient. A contact person, affiliated with the HCP team, will be appointed, and can be contacted in case AYAs or their family members might have any concerns about the upcoming transition, and transfer of care. AYAs are asked if they have a HCP confidant in the pediatric team, and if any, the appointed contact person will be the same person as the confidant. Next to a HCP contact person/confidant, AYAs will be asked to appoint one contact person of their trusted environment. All agreements made, and any beneficiary information will be documented in an individualized transition plan (ITP).
2. **Independent visit**: In between the transition consultation and joint consultation, at least one independent visit has to occur. If a parent/family member is present, they will be asked to leave part of the consultation. To guide the consultation, AYAs will have to fill in the Ready Steady Go-checklist beforehand ^24^, and the ITP will be re-evaluated and updated if necessary, to ensure AYAs still support their previously made decisions. Any concerns, questions or issues raised by the AYA during this independent visit will be addressed by the pediatric HCP team.
3. **Joint consultation**: The last consultation before the AYA will transfer to adult care, will be a joint consultation where pediatric and adult care HCPs are present. A new contact person at the adult care team will be appointed, and AYAs will be asked by whom of the pediatric care team they would like to be contacted for their feedback moment. To guide the consultation, AYAs will have to fill in the Ready Steady Go-checklist beforehand ^24^.
4. **Feedback moment**: Several months after their joint consultation, and preferably after their first consultation at the adult care department, AYAs will be contacted by the appointed pediatric HCP to evaluate the course of their transition and transfer to the adult healthcare team. To guide the feedback moment, AYAs will have to fill in the Ready Steady Go-checklist beforehand ^24^. Feedback will be shared among pediatric and adult care HCP, and if any concerns arise, pediatric and adult care HCPs will address these (referrals or extra consultations, if necessary).

**Figure 2. Four key moments of the TP.**

The ITP will guide AYA and HCP through the transition process. These plans will be set-up online, through the hospital portals, in collaboration with the ICT departments of the university hospitals. AYAs will gain access to their personalized ITP and be able to make changes if necessary. The content of the ITP can be found in the attachments.

## 3.3 │Setting and sampling

### Setting

A multicentric pre-test post-test study will be conducted at the Queen Mathilde Mother and Child Center of Antwerp University Hospital (UZA) and the Princess Elisabeth Pediatric Hospital of University Hospital Ghent (UZ Ghent). This study aims to implement the co-designed TP, as compared to usual care, over a total period of **16 months**, including a post-transfer assessment (see Figure 3). A local transition coordinator (TC) will facilitate the multicomponent TP to the IG over a course of approximately **9-15 months**, with three assessments (at baseline, moment of transfer and 4-months post-transfer), whereas the control group will receive usual care, with two assessments (at baseline and 4-months post-transfer). To avoid cross-contamination between IG and CG, the control period will commence three months prior to the IG. As soon as all participants of the CG have transferred to adult care, the intervention period will commence. To ensure treatment fidelity, the TC will provide the intervention using Standard Operating Procedures (SOPs).

**Figure 3. Design of pre-test post-test study.**

### Sampling and participants

Three study disease populations will be selected as a sample case for the broader spectrum of common chronic childhood-onset conditions, including AYAs with type 1 diabetes, asthma and/or obesity. These three populations are considered the target population for the TP, and therefore comprise the target population for the intervention study. Eligible participants for the intervention group (IG) are 17 years old; diagnosed with type 1 diabetes, asthma and/or obesity; who had at least one pediatric outpatient visit, prior to inclusion, in one of both hospitals within the past 12 months; are Dutch speaking; and are planned to transfer to adult care within the next 12 months. Eligible participants of the control group (CG) are aged between 17,5 and 18 years old, as the control period has a shorter duration (4 months vs. 12 months). Patients with severe mental, cognitive or neurological problems will be excluded. At the UZ Ghent, the target population will be type 1 diabetes patients. At the UZA, AYAs with asthma and obesity will be included.

To estimate the sample size of intervention study, the stepped rules of thumb were applied, considering a 90% powered main trial, a medium effect size (d<0.44) ^25^, and α=0.05. Feasibility studies are not confirmatory trials and thus should not be hypotheses-driven ^26^. These calculations indicated a required sample size of 15 patients per group (i.e., IG and CG). Hence, since patients will be recruited from three diagnostic groups, a total sample size of 90 participants is aimed for. Taking into account an attrition rate over the course of 16 months, and possible difficulties recruiting an adequate number of participants, a total number of 150 participants should be enrolled, resulting in at least 50 participants in each diagnostic group (n_IG_=25 and n_CG_=25), based on their primary care diagnosis (in case participants encounter multimorbidity).

For the process evaluation, all AYA participants of the IG, family members, and involved HCPs will be included for the quantitative assessment, resulting in a total sample size of (n=300). For the qualitative assessment, we will use a purposive subsample since it is not considered feasible to include all participants of the IG. Specifically, we will strive for variability of the participants’ characteristics in order to capture a breadth of experiences.

## 3.4 │Data collection

A comprehensive set of data will be collected to assess the TP’s preliminary impact in terms of PROMs and costs, next to process indicators.

### Effectiveness in terms of PROMS

As a TP primarily aims is to prepare patients for the transition to adulthood, the primary outcome of this study will comprise patients’ **transition readiness**, as measured using the Transition Readiness Assessment Questionnaire (TRAQ), version 6.0 ^27-29^. TRAQ is a 20-item instrument comprising two domains including self-management and self-advocacy, generating a score ranging from 0-53 ^29^. Secondary outcomes will comprise: **patient empowerment** (Gothenburg Young Persons Empowerment Scale (GYPES)) ^30^, **adherence rates** (Medication Adherence Questionnaire (MMAS) and Medication Adherence Report Scale (MARS-5)) ^31,32^, and psychosocial well-being including **health-related quality of life** (EuroQol-5D (EQ-5D-5L) ^33^, **anxiety and depression** (HADS) ^34^. Data on the health status will be collected as a part of standard care (from medical records). This includes: frequency of emergency admissions, missed consultations, and objective biomarkers (when relevant, e.g. HbA1c for diabetes patients, BMI for obesity patients, FEV_1_ for asthma patients). Patient-reported primary and secondary outcomes for the IG will be assessed at baseline (T_0_), moment of transfer (T_1_), and 4-months post-transfer (T_2_). The assessment of PROMS for the CG will take place at baseline (T_0_=moment of transfer) and at the 4-month post-transfer mark (T_1_).

### Process evaluation

Researchers, patients, families and HCPs, who participated in the TP, will be invited to participate in a process evaluation. Information will be collected during and at the end of the TP, using researcher observations, satisfaction questionnaires, and semi-structured interviews among all end users. Information on the timeline, structure, interventional components and outcomes assessed will be retrieved from the ITP. The use of various means of data collection allows for data triangulation and a comprehensive coverage of all program dimensions.

### Cost-effectiveness

Costs associated with program implementation (cost of additional staff (-time), i.e., physician, TC, nurses, psychologists, dieticians; cost of organizing multidisciplinary team meetings; cost of materials, e.g., printing material), as well as healthcare resource use will be collected. The latter will be based on codes available within the nomenclature of healthcare services from the NIHDI. Health effects collected to assess clinical effectiveness will be combined with EQ-5D outcomes to assess the health impact.

## 3.5 │Data analysis

### Clinical effectiveness analyses

Baseline characteristics will be summarized using descriptive statistics. A paired t-test will be performed as pre-posttest within the IG. Given that there will be no randomization and the IG and CG will not be completely similar in baseline characteristics, inverse probability treatment weighting (IPTW) will be used, creating a balance between relevant covariates that could confound the results. Weights will be calculated by inverting propensity scores, estimated in a regression analysis with whether or not participating in the TP as the dependent variable ^35^. After weighting, comparisons will be made between the IG and CG with independent t-tests. A multivariable regression analysis will be conducted to explore if the TP independently determined the set of outcomes. Significance levels will be set at p <0.05 and all tests will be performed two-sided.

### Process evaluation

A process evaluation will be conducted to assess the implementation of the TP and how patients, families and HCPs received it. This is important to entangle weaknesses and strengths of the TP and to better interpret the findings of the effect evaluation (i.e., why the TP was or was not successful). Information on several program dimensions will be evaluated, including: intervention fidelity (i.e., extent to which the delivered TP is consistent with the integrity and quality of the developed TP); dose (i.e., extent to which the TP was received by patients and families (dose received) and delivered by healthcare providers (dose completed)); reach (i.e., proportion and characteristics of patients that participates); context (i.e., contextual factors that improve or decrease TP uptake); and change (i.e., changes made by care team during study) ^36^. Quantitative information will be analyzed using descriptive statistics. Interviews will be audiotaped and transcribed verbatim. Qualitative data will be processed using a thematic approach via NVivo analysis software ^37^. At the end, information from the different data sources will be tabulated by the different program dimensions. Cross-checking of the reported results will be done, to investigate accuracy and consistency of the data. These analyses will give us a broad overview of the implementation process and how it was received by all parties, allowing us to adapt the TP accordingly, producing a modular well-functioning TP program that can serve as a prototype for wider implementation.

### Cost-effectiveness analyses

A health economic model will be developed in accordance with ISPOR guidelines on “Good Research Practices for Cost-Effectiveness Analysis Alongside Clinical Trials” to calculate the short and long term impact of the TP prototype, both in terms of costs and health effects ^38^. The TP will be compared with real-life routine clinical practice conditions. Trial outcomes included in the model will be focused on how patients feel and function. To assess the impact on survival or disease, intermediate endpoints that strongly relate to disease outcomes will be included. A weighted end-point (i.e., Quality adjusted life years (QALY)) will be used, accounting both for patients’ self-perceived health status and disease state. Cost calculations will be based on healthcare resource use collected during the intervention study as well as the operational cost of the TP. This will result in an incremental cost-effectiveness ratio (ICER) representing cost per quality adjusted life years (QALY). Future costs will be discounted at a rate of 3%, future benefits at a rate of 1.5%. To account for missing data, multiple imputation approaches will be used. Uncertainty surrounding the cost-effectiveness outcome will be handled appropriately. One-way sensitivity analyses will be performed to assess the impact of potential changes in input variables on the main outcome. Furthermore, a probabilistic sensitivity analysis using a bootstrapping approach will be performed to calculate the distribution around the ICER. A cost-effectiveness acceptability curve will be calculated allowing us to assess the possibility that the intervention is cost-effective using a range of willingness to pay thresholds.

# 4 │ETHICAL CONSIDERATIONS

The study entails a multicentric design, therefore, approvals will be sought simultaneously from the ethics committee of the respective participating centers. The Ethics Committee of UZA/UAntwerp will act as the central committee, and the Ethics Committee of UZ Ghent will act as the local committee. Additionally, approval from the BOC (UZ Ghent) will be sought. All studies proposed will be performed in accordance with the current ethical standards and privacy regulations (e.g., GDPR-regulations). Approval from appropriate ethical review committees will be obtained prior to study initiation. Formal written informed assent (<18y) or consent (≥18y) will be asked by every participant. When participants are 16 years or older, individual informed consent is considered sufficient, following the EU National Data Protection Authority guidelines ^39^. All participants will receive an information letter (submitted as attachments on the platform). Additionally, they will be asked to sign an informed consent or assent before participating in the study (submitted as attachments on the platform). The principles established in the Helsinki Declaration will be followed during the entire duration of the study. All participants will be informed that refusal to participate or to take part but subsequently withdraw from the study, would have no adverse effects on their current or future care. Data will be pseudonymized in codes developed by the principal investigator so confidentiality and privacy of personal data will be guaranteed. The data will be stored on an encrypted Sharepoint of UAntwerp (participants UZA) and the encrypted Sharepoint of UZ Ghent (participants UZ Ghent), with a double authentication process, for a period of 20 years.

# 5 │TIMELINE

**Figure 4. Gantt chart of the COCCOS feasibility study.**

# 6 │REFERENCES

1.	Perrin JM, Anderson LE, Van Cleave J. The Rise In Chronic Conditions Among Infants, Children, And Youth Can Be Met With Continued Health System Innovations. *Health Affairs*. 2014;33(12):2099-2105. doi:10.1377/hlthaff.2014.0832

2.	Turkel S, Pao M. Late consequences of pediatric chronic illness. *The Psychiatric clinics of North America*. 2007;30(4):819.

3.	Jin M, An Q, Wang L. Chronic conditions in adolescents. *Exp Ther Med*. Jul 2017;14(1):478-482. doi:10.3892/etm.2017.4526

4.	Cooley WC, Sagerman PJ. Supporting the Health Care Transition From Adolescence to Adulthood in the Medical Home. *Pediatrics*. 2011;128(1):182-200. doi:10.1542/peds.2011-0969

5.	Pediatrics AAo, Physicians AAoF, Medicine ACoP-ASoI. A consensus statement on health care transitions for young adults with special health care needs. *Pediatrics*. 2002;110(Supplement_3):1304-1306.

6.	Scal P. Transition for Youth With Chronic Conditions: Primary Care Physicians’ Approaches. *Pediatrics*. 2002;110(Supplement_3):1315-1321. doi:10.1542/peds.110.S3.1315

7.	White PH, Ardoin S. Transitioning wisely: improving the connection from pediatric to adult health care. *Arthritis & Rheumatology*. 2016;68(4):789-794.

8.	Prior M, McManus M, White P, Davidson L. Measuring the “Triple Aim” in Transition Care: A Systematic Review. *Pediatrics*. 2014;134(6):e1648-e1661. doi:10.1542/peds.2014-1704

9.	Van Walleghem N, Macdonald CA, Dean HJ. Evaluation of a systems navigator model for transition from pediatric to adult care for young adults with type 1 diabetes. *Diabetes Care*. Aug 2008;31(8):1529-30. doi:10.2337/dc07-2247

10.	Pape L, Ernst G. Health care transition from pediatric to adult care: an evidence-based guideline. *Eur J Pediatr*. May 2022;181(5):1951-1958. doi:10.1007/s00431-022-04385-z

11.	Goossens E, Bovijn L, Gewillig M, Budts W, Moons P. Predictors of Care Gaps in Adolescents With Complex Chronic Condition Transitioning to Adulthood. *Pediatrics*. Apr 2016;137(4)doi:10.1542/peds.2015-2413

12.	Fegran L, Hall EO, Uhrenfeldt L, Aagaard H, Ludvigsen MS. Adolescents' and young adults' transition experiences when transferring from paediatric to adult care: a qualitative metasynthesis. *Int J Nurs Stud*. Jan 2014;51(1):123-35. doi:10.1016/j.ijnurstu.2013.02.001

13.	Gabriel P, McManus M, Rogers K, White P. Outcome Evidence for Structured Pediatric to Adult Health Care Transition Interventions: A Systematic Review. *J Pediatr*. Sep 2017;188:263-269.e15. doi:10.1016/j.jpeds.2017.05.066

14.	Vaks Y, Bensen R, Steidtmann D, et al. Better health, less spending: redesigning the transition from pediatric to adult healthcare for youth with chronic illness. Elsevier; 2016:57-68.

15.	White PH, Cooley WC, Transitions Clinical Report Authoring G, American Academy Of P, American Academy Of Family P, American College Of P. Supporting the Health Care Transition From Adolescence to Adulthood in the Medical Home. *Pediatrics*. Nov 2018;142(5)doi:10.1542/peds.2018-2587

16.	Campbell F, Biggs K, Aldiss SK, et al. Transition of care for adolescents from paediatric services to adult health services. *Cochrane Database of Systematic Reviews*. 2016;(4)

17.	Bodenheimer T, Sinsky C. From triple to quadruple aim: care of the patient requires care of the provider. *Ann Fam Med*. Nov-Dec 2014;12(6):573-6. doi:10.1370/afm.1713

18.	Crowley R, Wolfe I, Lock K, McKee M. Improving the transition between paediatric and adult healthcare: a systematic review. *Arch Dis Child*. Jun 2011;96(6):548-53. doi:10.1136/adc.2010.202473

19.	Mazur A, Dembinski L, Schrier L, Hadjipanayis A, Michaud PA. European Academy of Paediatric consensus statement on successful transition from paediatric to adult care for adolescents with chronic conditions. *Acta Paediatrica*. 2017;106(8):1354-1357.

20.	Mora MA, Saarijärvi M, Moons P, Sparud-Lundin C, Bratt E-L, Goossens E. The scope of research on transfer and transition in young persons with chronic conditions. *Journal of Adolescent Health*. 2019;65(5):581-589.

21.	Janssens N, Van Wilder L, Van Hecke A, Van Hoorenbeeck K, Vanden Wyngaert K, De Smedt D, Goossens E. COCCOS study: Developing a transition program for adolescents with chronic conditions using Experience-Based Co-Design. A study protocol. *PLoS One*. 2024;

22.	Janssens N, Van Wilder L, Van Hecke A, Van Hoorenbeeck K, Vanden Wyngaert K, De Smedt D, Goossens E. COCCOS study: Developing a transition program for adolescents with chronic conditions using Experience-Based Co-Design. A study protocol. *PLoS One*. 2024;19(4):e0298571. doi:10.1371/journal.pone.0298571

23.	Wang C, Burris MA. Photovoice: Concept, methodology, and use for participatory needs assessment. *Health education & behavior*. 1997;24(3):369-387.

24.	Nagra A, McGinnity PM, Davis N, Salmon AP. Implementing transition: Ready Steady Go. *Arch Dis Child Educ Pract Ed*. Dec 2015;100(6):313-20. doi:10.1136/archdischild-2014-307423

25.	Cleverley K, Davies J, Allemang B, Brennenstuhl S. Validation of the Transition Readiness Assessment Questionnaire (TRAQ) 5.0 for use among youth in mental health services. *Child Care Health Dev*. Mar 2023;49(2):248-257. doi:10.1111/cch.13035

26.	Kunselman AR. A brief overview of pilot studies and their sample size justification. *Fertil Steril*. Jun 2024;121(6):899-901. doi:10.1016/j.fertnstert.2024.01.040

27.	Sawicki GS, Lukens-Bull K, Yin X, et al. Measuring the transition readiness of youth with special healthcare needs: validation of the TRAQ--Transition Readiness Assessment Questionnaire. *J Pediatr Psychol*. Mar 2011;36(2):160-71. doi:10.1093/jpepsy/jsp128

28.	Wood DL, Sawicki GS, Miller MD, et al. The Transition Readiness Assessment Questionnaire (TRAQ): its factor structure, reliability, and validity. *Acad Pediatr*. Jul-Aug 2014;14(4):415-22. doi:10.1016/j.acap.2014.03.008

29.	Johnson K, McBee M, Reiss J, Livingood W, Wood D. TRAQ Changes: Improving the Measurement of Transition Readiness by the Transition Readiness Assessment Questionnaire. *J Pediatr Nurs*. Jul-Aug 2021;59:188-195. doi:10.1016/j.pedn.2021.04.019

30.	Mora MA, Luyckx K, Sparud-Lundin C, et al. Patient empowerment in young persons with chronic conditions: Psychometric properties of the Gothenburg Young Persons Empowerment Scale (GYPES). *PLoS One*. 2018;13(7):e0201007. doi:10.1371/journal.pone.0201007

31.	Morisky DE, Green LW, Levine DM. Concurrent and predictive validity of a self-reported measure of medication adherence. *Med Care*. Jan 1986;24(1):67-74. doi:10.1097/00005650-198601000-00007

32.	Chan AHY, Horne R, Hankins M, Chisari C. The Medication Adherence Report Scale: A measurement tool for eliciting patients' reports of nonadherence. *Br J Clin Pharmacol*. Jul 2020;86(7):1281-1288. doi:10.1111/bcp.14193

33.	Kreimeier S, Greiner W. EQ-5D-Y as a Health-Related Quality of Life Instrument for Children and Adolescents: The Instrument's Characteristics, Development, Current Use, and Challenges of Developing Its Value Set. *Value Health*. Jan 2019;22(1):31-37. doi:10.1016/j.jval.2018.11.001

34.	Zigmond AS, Snaith RP. The hospital anxiety and depression scale. *Acta Psychiatr Scand*. Jun 1983;67(6):361-70. doi:10.1111/j.1600-0447.1983.tb09716.x

35.	Moons P. Propensity weighting: how to minimise comparative bias in non-randomised studies? *Eur J Cardiovasc Nurs*. Jan 2020;19(1):83-88. doi:10.1177/1474515119888972

36.	Saunders RP, Evans MH, Joshi P. Developing a process-evaluation plan for assessing health promotion program implementation: a how-to guide. *Health Promot Pract*. Apr 2005;6(2):134-47. doi:10.1177/1524839904273387

37.	*NVivo 14*. 2023.

38.	Ramsey SD, Willke RJ, Glick H, et al. Cost-effectiveness analysis alongside clinical trials II-An ISPOR Good Research Practices Task Force report. *Value Health*. Mar 2015;18(2):161-72. doi:10.1016/j.jval.2015.02.001

39.	Commission E. Can personal data about children be collected? Accessed 8/12/2023, <https://commission.europa.eu/law/law-topic/data-protection/reform/rights-citizens/how-my-personal-data-protected/can-personal-data-about-children-be-collected_en>

# **CONTACT**

**Principal Investigator**

Prof. dr. Eva Goossens, e-mail: eva.goossens@uantwerpen.be; eva.goossens@uza.be

**Local investigators at UZ Ghent/UGent**

- Prof. dr. Ann Van Hecke (local PI), e-mail: ann.vanhecke@uzgent.be; ann.vanhecke@ugent.be
- dr. Karsten Vanden Wyngaert, e-mail: karsten.vandenwyngaert@uzgent.be
- Prof. dr. Delphine De Smedt, e-mail: delphine.desmedt@ugent.be
- dr. Lisa Van Wilder, e-mail: lisa.vanwilder@ugent.be
- dra. Natwarin Janssens (joint PhD student), e-mail: natwarin.janssens@ugent.be

**Local investigators at UZA**

- Prof. dr. Kim Van Hoorenbeeck, e-mail: kim.vanhoorenbeeck@uantwerpen.be; kim.vanhoorenbeeck@uza.be
- dra. Natwarin Janssens (joint PhD student), e-mail: natwarin.janssens@uantwerpen.be
